# National Medical Dictionary

**Published:** 1890-06

**Authors:** 


### Book Reviews.

The National Medical Directory, including English, French, German, Italian and Latin Technical Terms used in Medicine and the Collateral Sciences, and a Series of Tables of Useful Data. By Jno. S. Billings, A. M., M. D., LL. D., Edin. and Harv. D. C. L. Oxon., etc. etc. With the collaboration of W. O. Atwater, M. D., Frank Baker, M. D., S. M. Burnett, M. D., W. T. Councilman, M. D., James M. Flint, M. D., J. A. Kidder, M. D., William Lee, M. D., R. Lorini, M. D., Washington Mathews, M. D., C. S. Minot, M. D., H. C. Yarrow, M. D. Lea Bros. & Co., Philadelphia. 1890.

Than Dr. Billings no more fit man for this important work could have been selected. His education and training have been such as to eminently prepare him for the work, which reflects much credit upon its gifted author. The profession needs no introduction to the writer, who is so well known through his previous contributions, *i. e.*, Index Catalogue and Index Medicus.

"The object of this Directory is to furnish to students and practioners of medicine a clear, concise definition of every medical term in current use in English, French, German and Italian medical literature, including the Latin terminology of all of these languages." That this object has been as thoroughly accomplished as possible with the constant addition of new terms, the work itself bears abundant evidence. The total number of words and phrases defined is 84,844.

The series of tables prefixed to the first volume of the work must prove of great benefit to students and practitioners of medicine, including, as they do, much very valuable information condensed from various sources. "They include a table of doses; of antidotes of the most common form of poisoning; of the inch and metre systems of numbering spectacle glasses; of thermometric scales; of the average dimensions of the fœtus at different ages; tables showing the average dimensions of the parts and organs of the adult human body, and of the weights of the organs of the human body," etc., etc., etc. The typography of the work is clear and distinct, and large enough to be read without fatiguing the eyes. The paper and binding are of superior quality.

This dictionary should occupy a place upon the shelves of every progressive medical man's library. F. W. M.

